# miR-338-3p inhibits epithelial-mesenchymal transition and metastasis in hepatocellular carcinoma cells

**DOI:** 10.18632/oncotarget.10138

**Published:** 2016-06-17

**Authors:** Jing-Song Chen, Li-Li Liang, Hong-Xu Xu, Fan Chen, Shun-Li Shen, Wei Chen, Lian-Zhou Chen, Qiao Su, Long-Juan Zhang, Jiong Bi, Wen-Tao Zeng, Wen Li, Ning Ma, Xiao-Hui Huang

**Affiliations:** ^1^ Department of Gastrointestinal Surgery, The First Affiliated Hospital of Guangzhou Medical University, Guangzhou, People's Republic of China; ^2^ Departments of Pediatrics, The First Affiliated Hospital of Sun Yat-Sen University, Guangzhou, China; ^3^ Departments of Laboratory, The First Affiliated Hospital, Sun Yat-Sen University, Guangzhou, China; ^4^ Departments of General Surgical Laboratory, The First Affiliated Hospital of Sun Yat-Sen University, Guangzhou, China; ^5^ Departments of Hepatobiliary Surgery, The First Affiliated Hospital of Sun Yat-Sen University, Guangzhou, China; ^6^ Animal Center, The First Affiliated Hospital, Sun Yat-Sen University, Guangzhou, China; ^7^ Department of Gastrointestinal, Hernia and Abdominal Surgery, The Sixth Affiliated Hospital, Sun Yat-Sen University, Guangzhou, China

**Keywords:** miR-338-3p, epithelial-mesenchymal transition, Snail1, N-cadherin, sonic hedgehog

## Abstract

Down-regulation of the miRNA miR-338-3p correlates with the invasive ability of hepatocellular carcinoma (HCC) cells. However, it is currently unclear whether down-regulation of miR-338-3p induces epithelial-mesenchymal transition (EMT), which may be the underlying mechanism governing HCC invasion. Here, we demonstrate that restoration of miR-338-3p expression via transfection of a miR-338-3p mimic reversed EMT and inhibited the motility and invasiveness of HCC cells. Conversely, silencing of endogenous miR-338-3p expression with a miR-338-3p-specific inhibitor induced EMT and enhanced HCC cell motility. Additionally, Snail1 (an upstream regulatory protein of EMT) and Gli1 (a key transcription factor in the sonic hedgehog (SHH) signaling pathway) expression was up-regulated in cells treated with the miR-338-3p inhibitor and down-regulated by the miR-338-3p mimic. Further analyses demonstrated that miR-338-3p inhibitor-induced EMT in HCC cells was blocked by treatment with a small interfering RNA (siRNA) targeting Snail1, that the SHH signaling pathway was required for both miR-338-3p inhibitor-induced EMT and up-regulation of Snail1, and that miR-338-3p targeted a sequence within the 3′-untranslated region of N-cadherin mRNA. Notably, miR-338-3p expression was significantly down-regulated in HCC samples from patients with metastases and was associated with poor metastasis-free survival rates. Lastly, correlations between the expression levels of miR-338-3p and E-cadherin, Smoothened (SMO), Gli1, Snail1, N-cadherin, and vimentin were confirmed in HCC xenograft tumors and HCC patient specimens. Our findings suggest that miR-338-3p suppresses EMT and metastasis via both inhibition of the SHH/Gli1 pathway and direct binding of N-cadherin. miR-338-3p is a potential therapeutic target for metastatic HCC.

## INTRODUCTION

Hepatocellular carcinoma (HCC) is one of the most common malignancies in China, and increasing incidences have been reported worldwide [1]. As with most other solid tumors, metastasis and local recurrence are responsible for the majority of HCC-related deaths [2]. Metastasis is a multistep process, and identifying the mechanisms responsible for cancer metastasis could assist in the development of novel therapeutic strategies. Accumulating evidence indicates that the epithelial-mesenchymal transition (EMT) is a first step toward metastasis [3]. EMT is characterized by a morphological change in cells from an epithelial-like to a mesenchymal-like appearance, which is accompanied by the down-regulation of E-cadherin, the up-regulation of mesenchymal markers (N-cadherin and vimentin), and increased cell invasiveness [4]. Recently, several studies have demonstrated that microRNAs (miRNAs) can act as critical modulators of EMT [5–8]. We previously reported that miR-338-3p expression is significantly down-regulated in both HCC patient tissues and cell lines and that this reduced expression correlates with higher histological staging in HCC patients [9]. Down-regulation of miR-338-3p has subsequently been reported in a number of other cancer types, including gastric cancer [10–11] and neuroblastoma [12]. In addition, our previous study demonstrated that miR-338-3p suppresses the invasiveness and metastasis of HCC cells by directly targeting the Smoothened (SMO) protein [13], a seven-pass transmembrane protein that is widely considered a marker of activation for the sonic hedgehog (SHH) signaling pathway [14]. Furthermore, Inaguma *et al.* (2011) demonstrated that activation of the SHH signaling pathway induces EMT through the inhibition of E-cadherin expression, leading to invasion and metastasis in pancreatic cancer cells [15]. Zhan *et al.* (2014) reported a positive correlation between Gli1 and Snail1 expression levels in progressive gastric cancer [16]. Snail1 is an upstream regulatory protein in EMT that reduces E-cadherin expression [17]. However, the correlation between the SHH signaling pathway and miR-338-3p expression in HCC is not fully understood.

While Tsuchiya *et al.* reported that up-regulation of miR-338-3p is necessary for the development of epithelial cell polarity in cancer cells [18], the relevance of miR-338-3p expression to EMT in HCC has yet to be investigated. In this study, we demonstrate that miR-338-3p both inhibited EMT through the SHH/Gli1/Snail1 signaling pathway and directly targeted the expression of N-cadherin in HCC cells undergoing EMT. Moreover, we investigated the correlation between miR-338-3p expression levels and the protein expression levels of SMO, Gli1, Snail1, E-cadherin, N-cadherin, and vimentin in HCC patients and an orthotopic xenograft HCC model in nude mice. Our results unveil a novel mechanism that links miR-338-3p expression to both the SHH signaling pathway and N-cadherin expression levels and provide a model that explains the aggressive characteristics of HCC.

## RESULTS

### The expression level of miR-338-3p inversely correlates with the EMT phenotype in HCC cells

**Figure 1:**
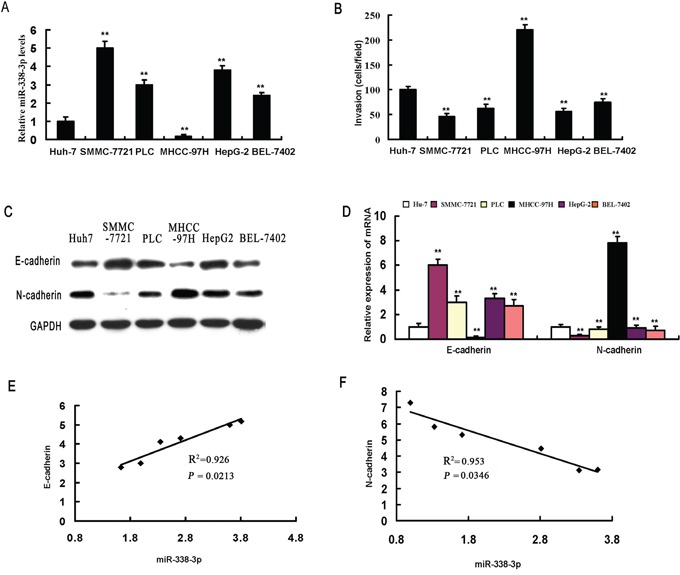
miR-338-3p expression levels correlate with invasive capacity and epithelial-mesenchymal transition (EMT) phenotype in hepatocellular carcinoma (HCC) cells **A.** miR-338-3p expression levels were measured in different HCC cell lines by real-time PCR. **B.** The invasive capacity of HCC cells was determined using invasion assays. Analysis of E-cadherin and N-cadherin expression levels in different HCC cell lines by **C.** western blot and **D.** real-time PCR. Correlation between the mRNA expression level of miR-338-3p and **E.** E-cadherin and **F.** N-cadherin. Data represent the results of three independent experiments. ^**^P < 0.01.

We first examined the effects of miR-338-3p expression on the invasive capacity and EMT phenotype of MHCC-97H, SMMC-7721, PLC, Huh7, HepG2, and BEL-7402 HCC cell lines and detected an inverse correlation between miR-338-3p expression and invasive potential (Figure 1A and 1B). In accordance with the invasive capability, E-cadherin was expressed at higher levels in less invasive cells (SMMC-7721), whereas highly invasive cells (MHCC-97H) expressed elevated levels of N-cadherin (Figure 1C and 1D). As such, there was a significant positive correlation between miR-338-3p expression and transcriptional expression of E-cadherin in HCC cells (Figure 1E) and an inverse correlation between miR-338-3p and N-cadherin expression levels (Figure 1F).

### miR-338-3p reverses EMT and reduces the invasiveness of HCC cells

**Figure 2:**
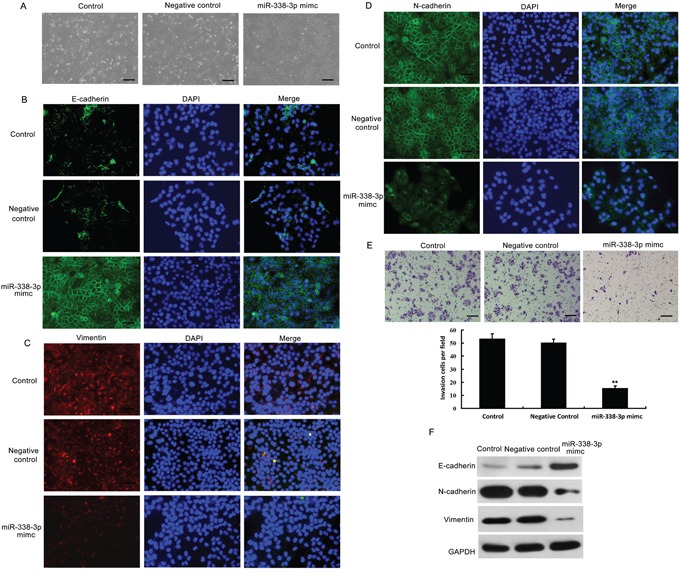
Effects of miR-338-3p overexpression on epithelial-mesenchymal transition (EMT) and invasiveness of hepatocellular carcinoma (HCC) cells **A.** Analysis of morphological changes in MHCC-97H cells after treatment with the miR-338-3p mimic or negative control miRNA by phase-contrast microscopy (original magnification, 100×). **B–D.** MHCC-97H cells transfected with the miR-338-3p mimic or negative control miRNA were re-plated on coverslips. After a 24-h incubation, cells were treated with (B) E-cadherin-, (C) vimentin-, or (D) N-cadherin-specific antibodies, stained with DAPI for visualization of the nuclei, and analyzed by fluorescence microscopy. The green and red signals represent staining for the corresponding protein, while DAPI-stained nuclei appear blue (original magnification, 200×). **E.** The invasive properties of the cells were analyzed by invasion assays using a Matrigel-coated Boyden chamber. Data are presented as the average number of cells that exhibited migration in each field from three different experiments (original magnification, 200×). **F.** Western blotting analysis was utilized to examine the expression levels of the epithelial protein E-cadherin and the mesenchymal markers N-cadherin and vimentin in MHCC-97H cells treated with the miR-338-3p mimic or negative control miRNA. GAPDH was used as a loading control. ^**^P < 0.01. Scale bars: 100 μm.

To further investigate the impact of miR-338-3p on EMT, we treated MHCC-97H cells with miR-338-3p mimics and examined the resulting phenotype. A dramatic morphological change was observed in MHCC-97H cells after treatment with miR-338-3p mimics; the spindle-like fibroblastic morphology was replaced by a typical cobblestone-like appearance (Figure 2A), suggesting that artificial overexpression of miR-338-3p induced mesenchymal-to-epithelial transition (MET), a reversal of the EMT process. To further assess whether miR-338-3p could reverse the EMT process, we analyzed the expression levels of epithelial and mesenchymal markers of EMT in miR-338-3p-treated and control cells. As shown in Figure 2B-2D and 2F, cells treated with the miR-338-3p mimic exhibited a normal epithelial phenotype compared to control cells, including enhanced expression of the epithelial marker E-cadherin and reduced expression of the mesenchymal markers N-cadherin and vimentin. We next utilized Matrigel-coated Boyden chamber invasion and wound healing assays (Supplementary Materials and Methods) to investigate the effects of miR-338-3p on cell invasion and motility. MHCC-97H (Figure 2E) and BEL-7402 (data not shown) cells treated with the miR-338-3p mimic exhibited significant decreases in invasiveness compared with the negative control-treated cell populations. Similar results were obtained via wound healing assay analyses using both MHCC-97H (Supplementary Figure 1) and BEL-7402 cells (data not shown). These data indicate that ectopic expression of miR-338-3p promoted MET, a reversal of EMT, in cells with a mesenchymal-like phenotype.

The effects of miR-338-3p on EMT were further investigated using miR-338-3p inhibitors. SMMC-7721 cells transfected with a miR-338-3p inhibitor acquired a spindle-shaped fibroblast-like morphology (Supplementary Figure 2A). Meanwhile, immunofluorescence and western blotting analyses showed markedly reduced expression of the epithelial protein marker E-cadherin and increased expression of vimentin, a mesenchymal-specific protein (Supplementary Figure 2B-2D, and 2F). Furthermore, SMMC-7721 cells underwent EMT and displayed a mesenchymal cell-like phenotype after treatment with the miR-338-3p inhibitor. The Matrigel chamber invasion experiment and wound healing assay demonstrated that inhibition of miR-338-3p in SMMC-7721 cells resulted in increased invasiveness (Supplementary Figure 2E) and cell mobility (Supplementary Figure 1), respectively, compared to negative control-treated cells. Similar results were obtained using PLC cells (data not shown). Together, our results suggest that miR-338-3p down-regulation is critical for the development of the mesenchymal morphology in HCC cells.

### Knockdown of Snail1 mitigates miR-338-3p-inhibitor-induced EMT

We next assessed whether miR-338-3p inhibition-mediated EMT correlated with the expression of Snail1. MHCC-97H cells treated with the miR-338-3p mimic exhibited decreased Snail1 mRNA and protein expression (Supplementary Figure 3A and 3B). Consistent with these results, treatment with the miR-338-3p inhibitor resulted in increased Snail1 mRNA and protein expression in SMMC-7721 cells (Supplementary Figure 3C and 3D).

**Figure 3:**
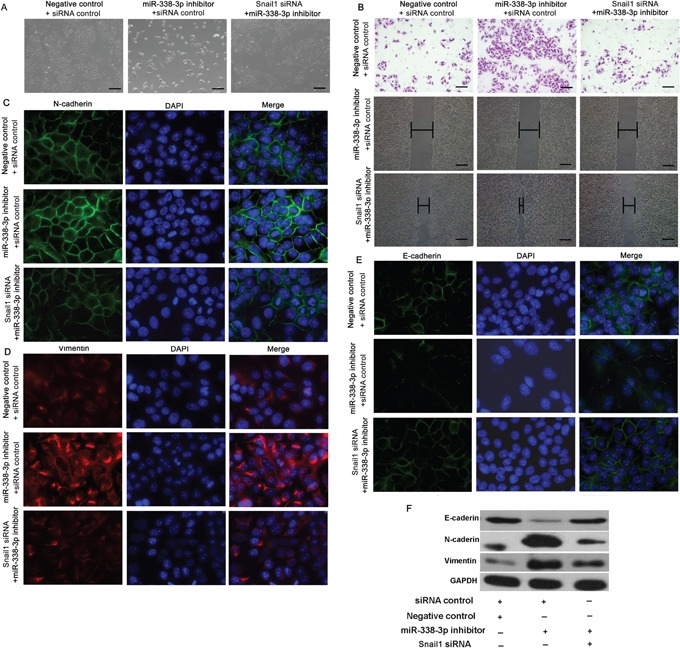
Effect of Snail1 expression on miR-338-3p-induced epithelial-mesenchymal transition (EMT) **A.** SMMC-7721 cells were treated with the miR-338-3p inhibitor or negative control miRNA and either Snail1 or control siRNA. Morphological changes in SMMC-7721 cells were observed by phase-contrast microscopy (original magnification, 100×). **B.** Quantification of the indicated invading cells as analyzed by Matrigel-coated Transwell assays. Wound healing assay analysis of SMMC-7721 cells transfected with the miR-338-3p inhibitor or Snail1 siRNA. Panels contain representative images of a single field under each condition at the beginning (*t* = 0) and end of the time course (*t* = 48 h) (original magnification, 200×). **C–D.** Immunofluorescence microscopy analysis of SMMC-7721 cells transfected with the miR-338-3p inhibitor or Snail1 siRNA showing (C) N-cadherin, (D) vimentin, and **E.** E-cadherin expression. DAPI stain was used to detect nuclei, and merged images are shown (original magnification, 400×). **F.** The expression levels of E-cadherin, N-cadherin, and vimentin were assessed in SMMC-7721 cells by western blot analysis. GAPDH was used as a loading control. Scale bars: 100 μm.

We subsequently assessed the contribution of Snail1 to EMT-related migration and invasion. SMMC-7721 cells were co-transfected with the miR-338-3p inhibitor or a negative control, and with Snail1 or control siRNA, and incubated for 48 h. The Snail1 siRNA partially reversed the miR-338-3p inhibitor-induced morphological changes and abrogated the observed increase in migration and invasion caused by the miR-338-3p inhibitor in SMMC-7721 cells (Figure 3A and 3B). Furthermore, the miR-338-3p inhibitor mediated marked increases in N-cadherin and vimentin expression in SMMC-7721 cells (Figure 3C-3D, 3F) that were reversed by the knockdown of Snail1 expression (Figure 3C-3D, 3F). Meanwhile, the miR-338-3p inhibitor caused a marked decrease in E-cadherin levels in SMMC-7721 cells that was rescued by treatment with the Snail1 miRNA (Figure 3E). Together, our results suggest that the effect of miR-338-3p on EMT is mediated by Snail1 signaling.

### The SHH signaling pathway is required for miR-338-3p inhibitor-induced EMT

While transfection of MHCC-97H cells with the miR-338-3p mimic resulted in a significant decrease in Gli1 protein and mRNA expression, transfection with the miR-338-3p inhibitor had the opposite effect (Supplementary Figure 3). Moreover, transfection of MHCC-97H cells with SMO-specific siRNA resulted in significant inhibition of both the miR-338-3p-mediated decrease in Snail1 and Gli1 expression and miR-338-3p inhibitor-mediated increase in Snail1 expression and induction of EMT (Supplementary Figure 4). SMO-knockdown cells also exhibited increased E-cadherin expression and decreased N-cadherin, Snail1, and vimentin expression, while Transwell, wound healing, and immunofluorescence assays confirmed that siRNA-mediated SMO knockdown prevented miR-338-3p inhibitor-induced EMT (Supplementary Figure 4A and 4C and Supplementary Figure 5A and 5B). These data suggest that down-regulation of miR-338-3p induces Snail1 expression and EMT in HCC cells via activation of the SHH/Gli1 pathway.

### Direct targeting of N-cadherin by miR-338-3p

**Figure 4:**
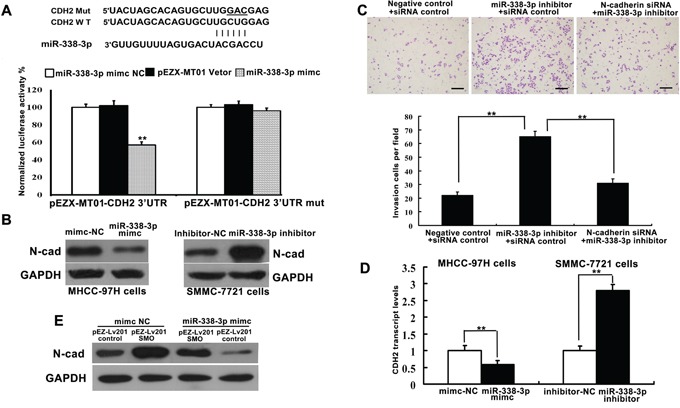
miR-338-3p directly and indirectly regulates the expression of N-cadherin **A.** Bioinformatics analysis of miR-338-3p was utilized to predict binding sites within the 3′-untranslated region (UTR) of the N-cadherin (CDH2) coding sequence. Luciferase reporter assay analysis indicated that miR-338-3p exerts a significant suppressive effect on the luciferase activity of pEZX-MT01-CDH2-3′-UTR-WT in MHCC-97H cells but has no effect on the mutant reporter vector pEZX-MT01-CDH2-3′-UTR-m. Data are representative of three independent experiments. **B, D.** Transient overexpression of miR-338-3p and knockdown of endogenous miR-338-3p by siRNA resulted in reduced and enhanced expression of N-cadherin protein and mRNA, respectively. **P < 0.01. **C.** SMCC-7721 cells were transfected with N-cadherin siRNA and/or the miR-338-3p inhibitor. Cell invasiveness was evaluated using a Transwell invasion chamber. **E.** Western blot analysis indicates that miR-338-3p indirectly suppresses N-cadherin protein expression. Scale bars: 100 μm.

The expression levels of N-cadherin in HCC remain controversial [19–20]. TargetScan analysis predicted a miR-338-3p binding site within the N-cadherin coding sequence (Figure 4A). SNAI1 is one of the transcriptional repressors involved in the regulation of the protein levels of N-cadherin [21]. Therefore, we examined whether miR-338-3p indirectly or directly inhibits expression of N-cadherin gene (*CDH2*) transcripts. MHCC-97H cells transfected with pre-miR-338-3p exhibited lower levels of CDH2 3′-UTR luciferase activity than the control, suggesting that miR-338-3p directly targets the 3′-UTR of CDH2 (Figure 4A). Moreover, while miR-338-3p overexpression significantly reduced the expression of N-cadherin mRNA and protein in MHCC-97H cells, transfection with the miR-338-3p inhibitor induced N-cadherin expression in SMMC-7721 cells (Figure 4B and 4D).

To determine whether N-cadherin knockdown could rescue the effects of miR-338-3p inhibition, SMMC-7721 cells were transfected with N-cadherin or control siRNA in the presence or absence of the miR-338-3p inhibitor. The observed increases in cell invasion and metastasis in cells treated with the miR-338-3p inhibitor were partly reversed by the N-cadherin siRNA treatment (Figure 4C). Meanwhile, SMMC-7721 cells transfected with both a plasmid encoding SMO without its 3′-UTR and the miR-338-3p mimic exhibited a decrease in the miR-338-3p-mediated inhibition of N-cadherin expression (Figure 4E). These results indicate that the observed miR-338-3p-dependent decrease in N-cadherin protein expression is partially mediated by the direct targeting of SMO by miR-338-3p. Thus, miR-338-3p represents a new class of miRNA that suppresses EMT and metastasis via regulation of the SHH/Gli1 pathway and direct targeting of N-cadherin.

### miR-338-3p inhibits EMT of HCC *in vivo*

**Figure 5:**
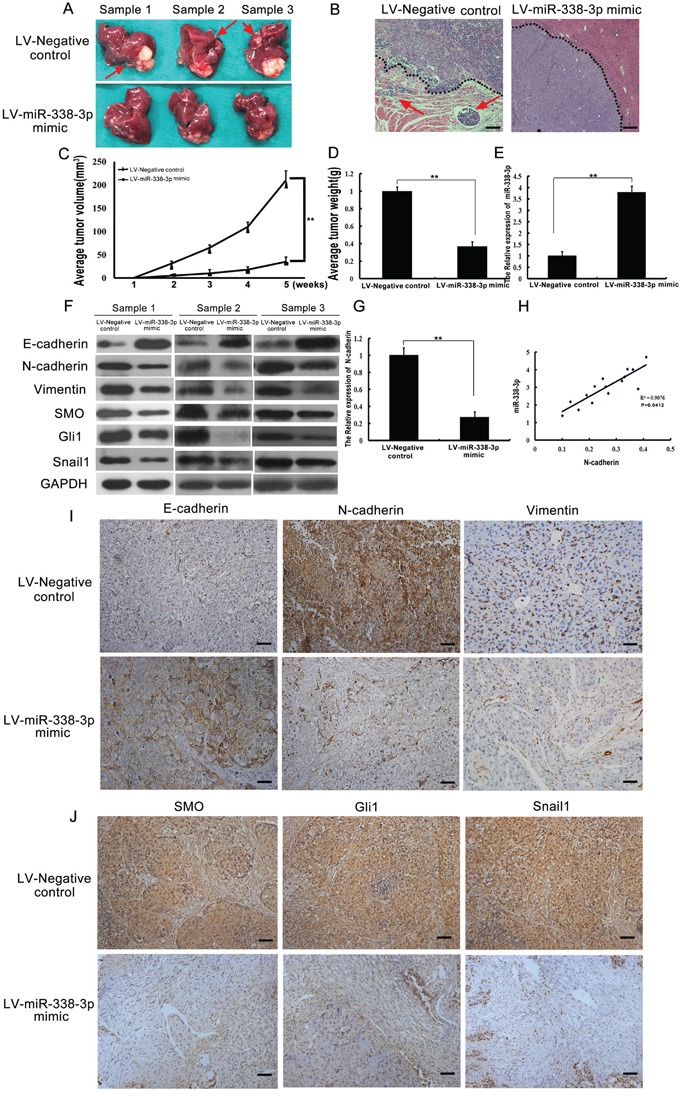
Analysis of miR-338-3p, Smoothened (SMO), Gli1, Snail1, E-cadherin, N-cadherin, and vimentin expression in an orthotopic xenograft hepatocellular carcinoma (HCC) model **A.** Representative images of livers from mice subjected to orthotopic implantation at week 4. Arrows indicate surface metastatic nodules. **B.** Representative images of intra-hepatic metastatic nodules stained with hematoxylin-eosin. The black dotted lines indicate the edges of the tumors. Tumors in the LV-Negative control group exhibited invasive edges, whereas those in the LV-miR-338-3p group had relatively smooth edges (original magnification, 40×). **C, D.** the average tumor volume and tumor weight between the groups. **E, G, H.** The expression of N-cadherin in the xenograft samples and the correlation between miR-338-3p and N-cadherin in HCC xenograft tumors. **F.** Western blotting analysis of E-cadherin, N-cadherin, vimentin, SMO, Gli1, and Snail1 expression levels in tumor samples. GAPDH was used as an internal control. All assays were performed in triplicate. **I.** Representative immunohistochemistry images of tumor tissues from different groups stained with E-cadherin-, N-cadherin-, and vimentin-specific antibodies. **J.** Immunohistochemical staining of SMO, Gli1, and Snail1 in tumor tissues from different groups (original magnification, 200×). Scale bars: 100 μm.

We further confirmed the effects of miR-338-3p expression on EMT *in vivo* using an HCC mouse model. The volume and weight of tumors from the LV-miR-338-3p group were significantly less than tumors of the LV-Negative control group (*P* < 0.05, Figure 5C and 5D). The livers of mice from the LV-miR-338-3p group contained significantly fewer micro-metastases than those from the LV-Negative control group (*P* < 0.05, Figure 5A). However, no metastatic nodules were found in the lungs of either the LV-miR-338-3p or the LV-Negative control mice. H&E staining of tumor sections detected fewer incidences of intrahepatic metastasis in the LV-miR-338-3p group than in the LV-Negative control group (Figure 5B). In addition, the tumors from the LV-Negative control group, which exhibited a typical EMT phenotype, had more invasive edges than those from the LV-miR-338-3p group (Figure 5B). Meanwhile, western blot analysis showed reduced levels of N-cadherin, vimentin, SMO, Gli1, and Snail1 protein expression in tumors from the LV-miR-338-3p group compared to those of the LV-Negative control group (Figure 5F). These results were subsequently confirmed by immunostaining assays (Figure 5I and 5J). Moreover, an inverse relationship between low N-cadherin expression and high miR-338-3p expression in the LV-miR-338-3p group was observed (Figure 5E, and 5H). These results confirm that miR-338-3p inhibits EMT in HCC via suppression of the SHH/Snail1 signaling pathway and N-cadherin *in vivo*.

### Significant correlation between low miR-338-3p expression in HCC tissues and metastasis in HCC patients

**Figure 6:**
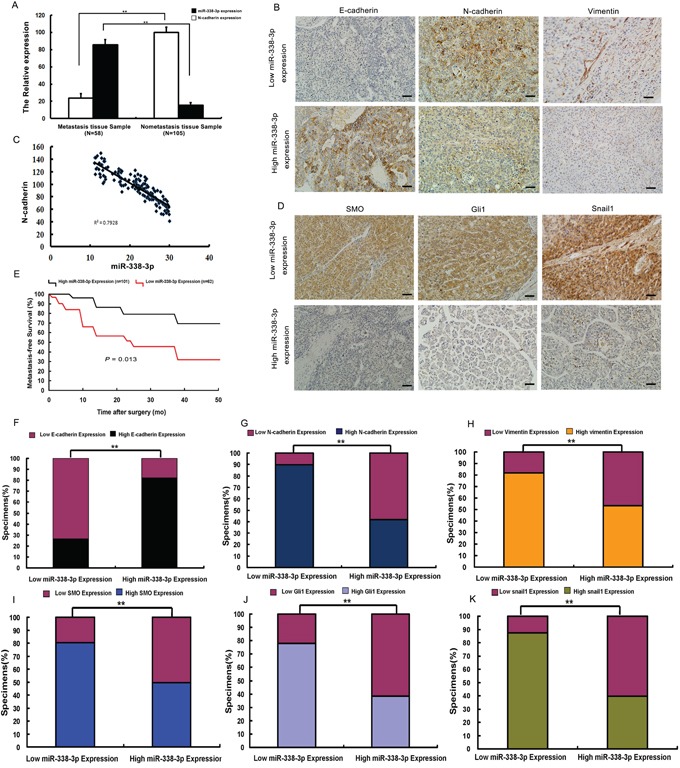
Analysis of miR-338-3p, Smoothened (SMO), Gli1, Snail1, E-cadherin, N-cadherin, and vimentin expression in hepatocellular carcinoma (HCC) patient tissues **A, C.** Quantitative reverse transcription PCR (qRT-PCR) analysis of the correlation between miR-338-3p and N-cadherin expression levels in HCC patients with (*n* = 58) and without metastasis (*n* = 105). U6 was used as an internal control. miR-338-3p expression levels were normalized to the mean value of all patients. **B.** miR-338-3p expression correlates with that of E-cadherin, N-cadherin, and vimentin in clinical HCC specimens. Representative images of immunohistochemically stained tissues are shown (original magnification, 200×). **E.** Kaplan-Meier curves of 163 HCC patients separated into two groups: high (greater than the mean; *n* = 101) and low (less than the mean; *n* = 62) miR-338-3p expression. miR-338-3p expression levels were used to calculate metastasis-free survival. The *P*-value was obtained by log-rank test. **D.** miR-338-3p expression levels correlate with those of SMO, Gli1, and Snail1 in clinical HCC specimens. Representative immunohistochemistry images are shown (original magnification, 200×). **F-K.** Percentage of specimens exhibiting low or high miR-338-3p expression and association of miR-338-3p expression with expression levels of E-cadherin, N-cadherin, vimentin, SMO, and Gli1, and Snail1 in clinical HCC specimens. **P < 0.01. Scale bars: 100 μm.

To further examine the relationship between miR-338-3p expression and N-cadherin, we measured miR-338-3p expression and N-cadherin levels in 163 HCC tissue specimens by qRT-PCR (Figure 6A). Based on the overall expression level of miR-338-3p, HCC specimens were divided into two groups: high and low miR-338-3p expression. While low levels of miR-338-3p expression were detected in 62 patients, 58 of which had metastasis and four which did not, high miR-338-3p expression was observed in 101 patients, none of which had metastasis. Notably, low miR-338-3p expression levels were significantly associated with poor metastasis-free survival (Figure 6E). In addition, the results showed an inverse relationship between high N-cadherin expression and low miR-338-3p expression in HCC patients with metastasis; N-cadherin expression in specimens of the high miR-338-3p expression group was significantly less than in the low miR-338-3p expression group (*P* < 0.05, Figure 6A and 6C). These findings suggest a significant correlation between low miR-338-3p expression and HCC metastasis.

### Correlation between expression of miR-338-3p and expression of SMO, Gli1, E-cadherin, and vimentin in HCC patients

Next, we assessed whether there was an association between the expression level of miR-338-3p and that of SMO, activated Gli1, Snail1, and EMT biomarkers in HCC patients. Quantitative RT-PCR analysis showed higher levels of SMO, Gli1, Snail1, N-cadherin, and vimentin expression in patients with metastasis, whereas elevated protein expression of E-cadherin was detected in patients without metastasis (Supplementary Figure 6). Moreover, tumors that exhibited high levels of miR-338-3p demonstrated low levels of SMO, Gli1, Snail1, N-cadherin, and vimentin but high levels of E-cadherin. Contrasting results were obtained from tissues that exhibited low miR-338-3p expression (Figure 6B, and 6F-6K). These findings show that there was an inverse correlation between the expression levels of SMO, Gli1, Snail1, N-cadherin, and vimentin and that of miR-338-3p in HCC tumor samples. Together, these results further suggest that down-regulation of miR-338-3p in HCC is associated with the up-regulation of SMO, the activation of the SHH signaling pathway and Snail1, and ultimately, the promotion of metastasis in HCC through induction of EMT.

## DISCUSSION

Although miR-338-3p has been shown to suppress cell migration and invasion by targeting SMO [13], and reduced expression of miR-338-3p is a frequent event in a variety of cancers [10–12], the involvement of miR-338-3p in EMT has yet to be investigated. In this study, we characterized miR-338-3p as a novel EMT inhibitor by demonstrating that down-regulation of miR-338-3p induced EMT and increased cell invasion in HCC cell lines. Decreased expression of miR-338-3p resulted in the activation of the SHH/Snail1 pathway, increased N-cadherin expression, and reduced inhibition of EMT. Furthermore, we showed that down-regulation of miR-338-3p was linked to increased metastasis and shortened metastasis-free survival in HCC patients.

EMT initiates cancer cell dissemination and leads to metastasis, and increasing evidence indicates that miRNAs play important roles in this process [21]. Indeed, dysregulation of certain miRNAs has been found to play an important role in the initiation of EMT. Zhang *et al*. (2014) reported that miR-148a suppresses EMT in hepatoma cells by targeting the Met/Snail signaling pathway [22].

Consistent with our previous work, recent studies from other laboratories have demonstrated an essential role for miR-338-3p in various cancers [23]. For example, miR-338-3p inhibits neuroblastoma invasion and migration by targeting PREX2a [10]. However, the effects of miR-338-3p on EMT have not yet been investigated. In the present study, we demonstrated that down-regulation of miR-338-3p induced EMT, while overexpression of miR-338-3p reversed EMT in HCC cells. We also found that the expression of miR-338-3p was inversely correlated with invasive capacity and an EMT phenotype in HCC cells. Therefore, our data confirm that miR-338-3p expression levels have a significant impact on EMT in HCC.

A critical step in EMT is the down-regulation of E-cadherin [24]. The transcription factor Snail1, which is a central regulator of EMT, directly suppresses and enhances the transcriptional expression of E-cadherin and N-cadherin, respectively [17]. While Cai *et al*. (2012) reported that aberrant Snail1 expression is related to HCC [25], an association between Snail1 and miR-338-3p expression in HCC has yet to be demonstrated. Our data indicate that Snail1 expression was inhibited by a miR-338-3p mimic and enhanced by a miR-338-3p inhibitor, while knockdown of Snail1 expression reversed the effects of miR-338-3p inhibition on EMT. In addition, we found that miR-338-3p expression was closely associated with that of Snail1 in HCC samples. Together, these results suggest that decreased miR-338-3p induces EMT in HCC cells via the elevated expression of Snail1.

The SHH signaling pathway is another key regulatory pathway that induces EMT in HCC [26]. Previous studies demonstrated that activation of this pathway directly induces EMT via fibroblast growth factor and leads to invasion and metastasis through the up-regulation of vimentin and the down-regulation of E-cadherin [27, 28, 29]. SMO up-regulation, which we previously demonstrated is inhibited via miR-338-3p binding to the SMO 3′-UTR [15], activates the SHH pathway [30]. Furthermore, transcriptional expression of Snail1 is directly induced by Gli1 [31]. In the present study, we showed that there was a concurrent increase or decrease in Gli1 expression when miR-338-3p was silenced or overexpressed, respectively. Subsequently, we demonstrated that the effects of down-regulation of miR-338-3p, including increases in Snail1 expression, cell migration, cell invasion, and EMT, were abolished by siRNA-mediated SMO depletion. In addition, we revealed that miR-338-3p expression is inversely correlated with metastatic recurrence in HCC patients, while orthotopic tumor metastasis assays demonstrated that stable expression of miR-338-3p in MHHC-97H cells suppresses the incidence of intrahepatic metastasis *in vivo* compared with the LV-Negative control group. Lastly, we observed a negative correlation between the expression level of miR-338-3p and that of N-cadherin, SMO, Gli1, Snail1, and vimentin in both the orthotopic liver xenograft mouse model and in human HCC tissues. Therefore, our data indicate that upon down-regulation of miR-338-3p, the SHH signaling pathway promotes Snail1 expression to induce EMT in HCC cells. N-cadherin has been shown to be involved in EMT and cancer progression with respect to metastasis [32]. In addition to affecting the SHH/Gli1/Snail1 pathway, our results show that miR-338-3p directly affected the expression of the N-cadherin protein to inhibit HCC EMT.

In conclusion, the regulation of miR-338-3p expression plays an important role in the pathogenesis of HCC. Specifically, down-regulation of this miRNA promotes N-cadherin expression and SMO-mediated activation of the SHH/Snail1 pathway, leading to EMT. The discovery of this novel function and molecular mechanism for decreased miR-338-3p expression in cancer provides important insight into HCC progression and metastasis and could assist in the development of novel therapeutic strategies for HCC.

## MATERIALS AND METHODS

### Patients and samples

A total of 163 patients who underwent hepatectomy for HCC at the First Affiliated Hospital of Sun Yat-sen University (Guangzhou, China) between January 2004 and December 2008 were enrolled in this study. The study protocol was reviewed and approved by the Ethics Committee of the First Affiliated Hospital of Sun Yat-sen University and conformed to the ethical guidelines of the 1975 Declaration of Helsinki. Clinical samples were collected from patients after obtaining written informed consent. Surgical tissue samples were immediately frozen at −80°C and embedded in paraffin. All patient diagnoses of primary HCC were confirmed by pathology, and none of the patients received any pre-operative cancer treatments. All patients were followed-up after surgery until July 5, 2012; the median follow-up was 16.8 months. Patient clinicopathological characteristics are included in Supplementary Table 1. Metastasis-free survival was defined as the interval between surgery and metastasis. By comparing the median value of miR-338-3p expression among the cancer tissue samples analyzed, patients were subcategorized as exhibiting either low (*n* = 81) or high miR-338-3p expression (*n* = 82).

### Cell lines and transfection

Six human HCC cell lines of varying metastatic potential (MHCC-97H, SMMC-7721, PLC, Huh7, HepG2, and BEL-7402) were used in this study [15]. MHCC-97H and SMMC-7721 cells were transfected with miRNA and siRNA molecules using Lipofectamine 2000 reagent (Invitrogen, Carlsbad, CA, USA) according to the manufacturer's instructions. More detail is provided in the Supplementary Materials and Methods section.

### Generation of stable cell lines overexpressing miR-338-3p

Lentiviral pEZX-MR03 plasmids containing miR-338-3p or negative control miRNA precursor sequences (termed LV-miR-338-3p and LV-miR-Negative control, respectively) were purchased from GeneCopoeia (Rockville, MD, USA). Plasmids were transfected into 293Ta cells with EndoFectin Lenti transfection reagent (GeneCopoeia) according to the manufacturer's instructions. After a 48-h incubation, supernatants containing lentiviral particles were collected and filtered by centrifugation (500 × *g* for 10 min). MHCC-97H cells were then transduced with lentiviruses expressing miR-338-3p or the negative control sequence. To select stably transduced cells, puromycin (2 μg/ml) was added to the culture medium at 48 h post-transfection, and the cells were cultured for 14 days. Transfection efficiency was monitored and examined by western blotting. Selected clones were then cultured for use in animal studies.

### Animal xenograft tumor model

Six-week-old female BALB/c nude mice were obtained from the Animal Experimental Center of the First Affiliated Hospital of Sun Yat-sen University. All procedures were approved by the Animal Care Committee of the First Affiliated Hospital of Sun Yat-Sen University, and animals received humane care according to the criteria outlined in the “Guide for the Care and Use of Laboratory Animals” prepared by the National Academy of Sciences and published by the National Institutes of Health (NIH publication 86-23 revised 1985). Equal numbers (1 × 10^7^) of MHCC-97H or control cells were subcutaneously injected into the right lateral flanks of the nude mice. To establish orthotopic implantation mice, subcutaneous tumors were removed after 3 weeks, cut into pieces (1 mm^3^), and implanted into the left lobes of the livers of a second group of nude mice. Two groups of 15 mice each were then treated with MHCC-97H cells infected with either LV-Negative control or LV-miR-338-3p lentivirus via tail vein injection. Tumor size was measured weekly. Mice were sacrificed after 4 weeks, and tumor volume was estimated. The lungs and livers were removed from the mice *and* embedded in paraffin. The paraffin-fixed tissues were serially sectioned and stained with hematoxylin-eosin (H&E) to identify metastatic nodules. Furthermore, tissues were subjected to immunostaining for detection of E-cadherin, N-cadherin, vimentin, Gli1, SMO, and Snail1 expression. Lastly, the tumor tissues were used for western blotting or qRT-PCR analyses.

### Statistical analysis

Experimental data are presented as the means ± standard deviations (SD). Comparisons between results were performed using two-tailed Student's t-tests with SPSS 13.0 software for Windows (SPSS Inc., Chicago, IL, USA). Qualitative data were analyzed by chi-squared (χ^2^) test, while the log-rank test was used to determine the statistical significance of differences in metastasis-free survival curves between the patients who exhibited distinct levels of protein expression. Spearman's correlation test was used to analyze the correlation between miR-338-3p expression and that of EMT-related proteins. *P*-values < 0.05 were considered statistically significant for all tests.

## SUPPLEMENTARY MATERIALS FIGURES AND TABLES



## Abbreviations

EMT: epithelial-mesenchymal transition
miRNA: micro RNA
HCC: hepatocellular carcinoma
SHH: sonic hedgehog
siRNA: small interfering RNA
SMO: Smoothened
DMEM: Dulbecco's modified Eagle's medium
FBS: fetal bovine serum
qRT-PCR: quantitative reverse transcription polymerase chain reaction
H&E: hematoxylin-eosin
MET: mesenchymal-epithelial transition
