# Supplementary material for: miR-338-3p inhibits epithelial-mesenchymal transition and metastasis in hepatocellular carcinoma cells
Source: Oncotarget. 2016 Jun 17;8(42):71418–29. doi: 10.18632/oncotarget.10138 (PMC5641059; doi:10.18632/oncotarget.10138)
Supplement: Supplementary file 1 [file oncotarget-08-71418-s001.pdf]

miR-338-3p inhibits epithelial-mesenchymal transition and metastasis in hepatocellular carcinoma cells

SUPPLEMENTARY MATERIALS AND METHODS

Cell lines and transfection

MHCC-97H and SMMC-7721 cells were transfected with 50 nM of the miR-338-3p mimic or negative control miRNA (RiboBio, Co., Ltd., Guangzhou, China), and 50 nM of the miR-338-3p inhibitor or the negative control miRNA (RiboBio), respectively, using Lipofectamine 2000 reagent (Invitrogen, Carlsbad, CA, USA), according to the manufacturer's instructions. Meanwhile, the small interfering RNA (siRNA) specific to SMO and the corresponding control siRNA (RiboBio) were transfected into SMMC-7721 cells, as described previously [1]. The three siRNA duplex oligonucleotides specific to human Snail1 and N-cadherin mRNA utilized in this study were also synthesized by RiboBio. For our analyses, we chose to utilize Snail1 siRNA1 and N-cadherin siRNA1 as they effectively inhibited endogenous Snail1 and N-cadherin expression, respectively. For these experiments, SMMC-7721 cells were transfected with 100 nM of the Snail1 siRNA1, the N-cadherin siRNA1, or the control siRNA using Lipofectamine 2000 reagent. After the aforementioned treatments, morphological changes in HCC cells were monitored by inverted phase-contrast microscopy, and siRNA knockdown efficiencies were confirmed by quantitative reverse transcription PCR (qRT-PCR) and western blotting analyses. All RNA oligoribonucleotides used in this study are listed in Supplementary Table 2.

RNA extraction and qRT-PCR

Total RNA, including small RNAs, was extracted from tissues or cells using TRIzol reagent (Invitrogen), according to the manufacturer's protocol. Complementary DNA (cDNA) was synthesized and qRT-PCR was performed as previously described [2]. The primers used for detection of human SMO, Gli1, Snail1, N-cadherin, E-cadherin, vimentin, and β-actin expression were reported previously [3]. A TaqMan miRNA assay kit (Applied Biosystems, Waltham, MA, USA) was used for the detection of mature miR-338-3p expression, as described previously [4]. The expression levels of target genes were normalized to that of U6 and β-actin for miRNAs and mRNAs assays, respectively. All primers used in this study are listed in Supplementary Table 2.

Immunofluorescent staining

The cells were cultured on coverslips, fixed with 4% paraformaldehyde (Sigma, St Louis, MO,), permeabilized by 1% sodium dodecyl sulfate, incubated in a blocking buffer (1% bovine serum albumin, 0.25% Triton X-100 in phosphate-buffered saline, pH 7.4), and then incubated with primary antibodies against E-cadherin, N-cadherin or vimentin overnight at 4°C. This was followed by incubation with rhodamine-conjugated or FITC-conjugated goat antibodies against rabbit or mouse IgG (Jackson ImmunoResearch Laboratories, West Grove, PA) and nuclear counter-staining with 4,6-diamidino-2-phenylindole (DAPI). The fluorescent images were obtained using the AxioVision Rel. 4.6 computerized image analysis system (Carl Zeiss Meditec AG, Jena, Germany).

Cell migration and invasion assays

The cell migration and invasion assays were performed as previously described [5]. Briefly, for the migration assay, at 24 h after transfection, cells (5×10^4/well) were seeded into the upper chamber of a 24-well Transwell insert (Corning, New York, NY) in serum-free medium. After 24-h incubation, the cells on the upper surface of the filter were wiped off with a cotton swab, and the cells that had invaded into the lower surface of the filter were fixed with 4% paraformaldehyde, stained with crystal violet, and counted under a microscope at 200× magnification. The cellular invasion potential was determined by these same procedures but with a Matrigel coating on the filter.

Wound healing assay

The transfected cells were seeded on to 24-well plates and grown in Dulbecco's modified Eagle's medium (DMEM). After 48-h incubation and once the culture reached approximately 90% confluence, an artificial homogenous wound was created on the monolayer with a sterile 200-μL plastic micropipette tip. After scratching, the cells were washed twice with PBS and incubated for additional 48 h. The images of cells migrating into the wound were observed at indicated times and captured with an inverted microscope under 100× power (BX50; Olympus, Tokyo, Japan).

Western blotting analysis

Western blotting was conducted according to a standard method described previously [1]. In brief, the total protein was extracted from tissues or cells, and protein concentration was measured with a Bio-Rad protein assay kit (Bio-Rad, Hercules, CA,). The samples with equal amounts of protein were separated by 10% sodium dodecyl sulfate polyacrylamide gel electrophoresis (SDS-PAGE) and transferred to polyvinylidene difluoride (PVDF) membranes (Millipore, Bedford, MA,). The membranes were incubated with primary antibodies against E-cadherin (Cell Signaling Technology, West Grove, PA) at 1:800, N-cadherin (Cell Signaling Technology) at 1:800, Gli1 (Santa Cruz Biotechnology, Santa Cruz, CA) at 1:500, SMO (Santa Cruz Biotechnology) at 1:500, Snail1 (Cell Signaling Technology) at 1:800, vimentin (Santa Cruz Biotechnology) at 1:500, and GAPDH (Santa Cruz Biotechnology at 1:1000 overnight at 4°C, followed by washes with TBS and incubation with the corresponding secondary HRP-conjugated antibodies (Santa Cruz Biotechnology). Protein bands were detected with an enhanced chemiluminescence (ECL) kit (Pierce, Rockford, IL) and exposure to X-ray film.

Immunohistochemistry

Immunohistochemistry was performed as described previously [5]. The degree of immunohistochemical staining was examined and scored independently by two observers. The staining intensity was scored according to the following criteria: no staining (0), weak staining (1), moderate staining (2), and strong staining (3), as described previously [2]. The cut-off values for high and low expression of indicated proteins were chosen based on a measurement of heterogeneity using the log-rank test. Images were captured at 200× magnification using the AxioVision Rel. 4.6 computerized image analysis system (Carl Zeiss).

Luciferase reporter assay

Cadherin gene CDH2 3′-untranslated region (CDH2 3′-UTR) luciferase reporter constructs were generated by cloning either the wild type or mutant 3′-UTR sequence of the CDH2 coding sequence into the pEZX-MT01-reporter construct (GeneCopoeia) downstream of the luciferase gene using the primers listed in Supplementary Table S2.

PCR products were cloned into PEZX-MT01 at the same site, and the identities of the resulting clones (pEZX-MT01-CDH2 3′-UTR-WT and pEZX-MT01-CDH2-3′-UTR-m) were confirmed by sequencing analysis. To confirm binding between miR-338-3p and the 3′-UTR of CDH2, the mutant CDH2 3′-UTR-m reporter was transfected into MHCC-97H cells together with pre-miR-338-3p. At 48 h after transfection, cells were harvested and analyzed using a Dual-Luciferase Reporter Assay System Kit (Promega, Madison, WI, USA). For these analyses, firefly luciferase activity was normalized to *Renilla* luciferase activity. The cDNA of human SMO (lacking the 3′-UTR) was PCR amplified and inserted into pEZ-Lv201 expression vector using the primers listed in Supplementary Table 2. All experiments were performed twice and three experimental replicates were included per experiment.

REFERENCES

1. Huang XH, Chen JS, Wang Q, Chen XL, Chen LZ, Li W, *et al*. miR-338-3p suppresses invasion of liver cancer cell by targeting smoothened. J Pathol 2011; 225:463-472.
2. Chen JS, Wang Q, Fu XH, Huang XH, Chen XL, Cao LQ, *et al*. Involvement of PI3 K/PTEN/AKT/mTOR pathway in invasion and metastasis in hepatocellular carcinoma: association with MMP-9. Hepatol Res 2009; 39:177–186. 23.
3. Chen JS, Li HS, Huang JQ, Zhang LJ, Chen XL, Wang Q, *et al*. Down-regulation of Gli-1 inhibits hepatocellular carcinoma cell migration and invasion. Mol Cell Biochem 2014; 393:283-291.
4. Huang XH, Wang Q, Chen JS, Fu XH, Chen XL, Chen LZ, *et al*. Bead-based microarray analysis of microRNA expression in hepatocellular carcinoma: miR-338 is downregulated. Hepatol Res 2009; 39:786-794.
5. Chen JS, Wang Q, Fu XH, Huang XH, Chen XL, Cao LQ, *et al*. Involvement of PI3 K/PTEN/AKT/mTOR pathway in invasion and metastasis in hepatocellular carcinoma: association with MMP-9. Hepatol Res 2009; 39:177–186.

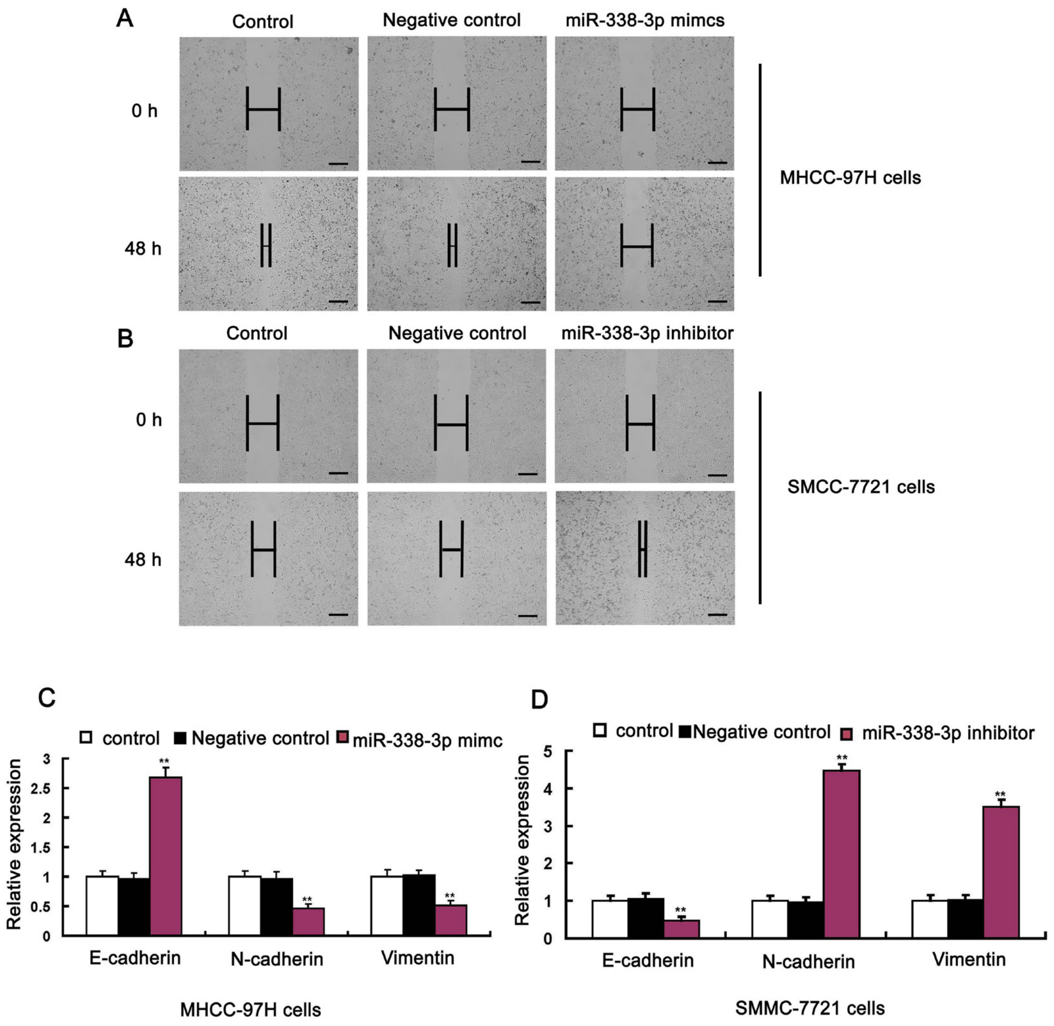

Supplementary Figure S1: The effects of miR-338-3p on migration of HCC cells and the mRNA levels of EMT markers. **A.** Wound healing assay of MHCC-97H cells transfected with negative control or miR-338-3p mimic. Representative pictures of one field at the beginning ($t = 0$) and at the end of the recording ($t = 48$ h) in each condition are shown. **B.** Wound healing assay of SMMC-7721 cells transfected with negative control or miR-338-3p inhibitor. Representative pictures of one field at the beginning ($t = 0$) and at the end of the recording ($t = 48$ h) in each condition are shown. **C.** Real-time PCR results showing relative levels of E-cadherin, N-cadherin, and vimentin in MHCC-97H with miR-338-3p mimic or negative control. **D.** Real-time PCR results showing relative levels of E-cadherin, N-cadherin and vimentin in SMMC-7721 with miR-338-3p inhibitor or negative control. Scale bars: 100 μm.

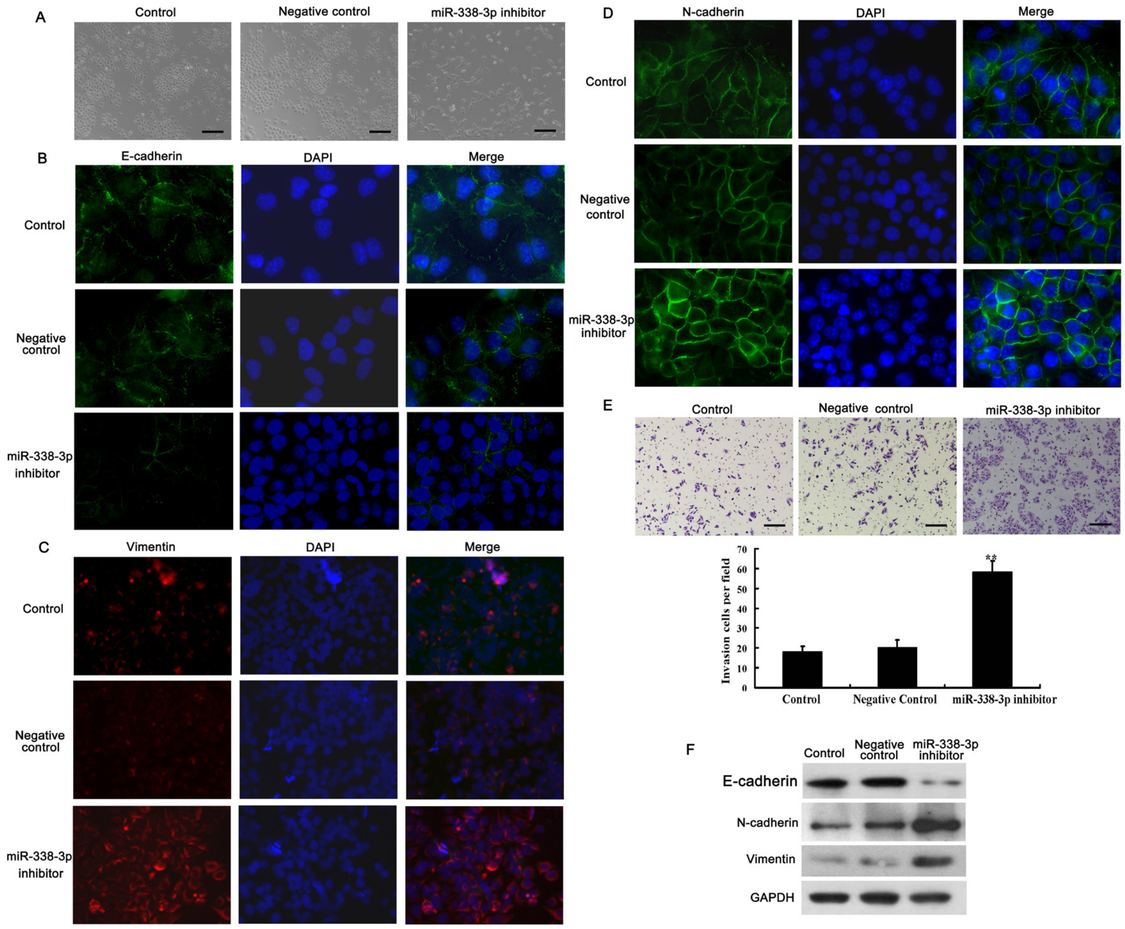

Supplementary Figure S2: Down-regulation of miR-338-3p induced EMT and enhanced the invasiveness of HCC cells. **A.** Morphological changes in SMMC-7721 cells treated with miR-338-3p inhibitor or miRNA negative control are shown by phase-contrast microscopy. Original magnification, 100×. **B, C, D.** SMMC-7721 cells treated with miR-338-3p inhibitor or miRNA negative control were re-plated on coverslips. After an additional 24 hours, cells were stained for E-cadherin, N-cadherin, vimentin, and DAPI and analyzed by microscopy. The green signal and the red signal represent staining for the corresponding protein, and DAPI staining was used to detect nuclei. Original magnification, 200×. **E.** The invasive properties of the cells were analyzed by an invasion assay using a Matrigel-coated Boyden chamber. Migrated cells were plotted as the average number of cells per field of view from 3 different experiments, as described in Methods. Original magnification, 200×. **F.** Expression of the epithelial protein E-cadherin and the mesenchymal markers N-cadherin and vimentin in MHCC cells treated with miR-338-3p inhibitor or miRNA negative control were detected by Western blotting. GAPDH was used as a loading control. $^{**}P < 0.01$. Scale bars: 100 µm.

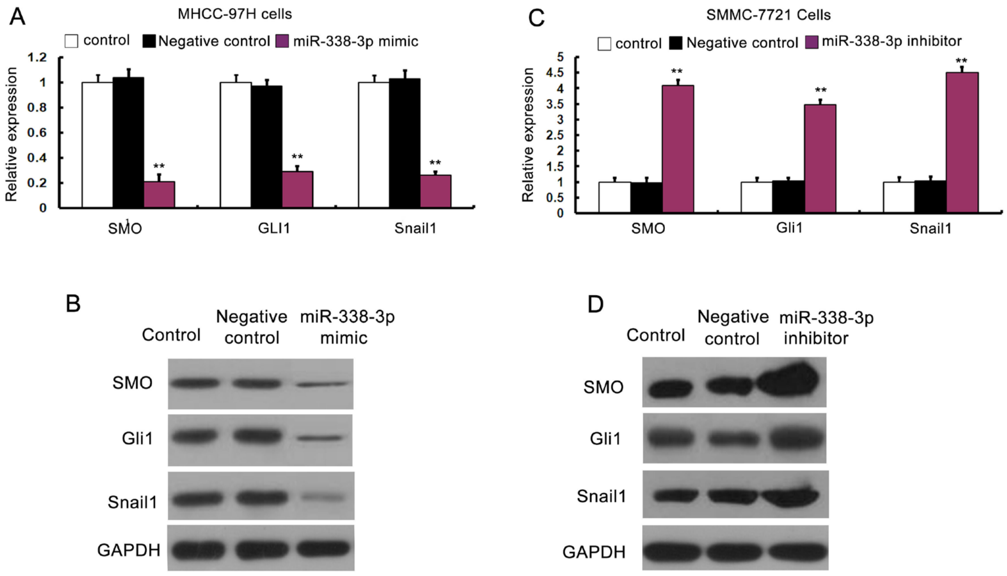

Supplementary Figure S3: Effect of miR-338-3p on Snail1 expression and the sonic hedgehog (SHH) pathway in hepatocellular carcinoma (HCC) cells. A, C. Quantitative reverse transcription PCR (qRT-PCR) analysis of the relative expression levels of Smoothened (SMO), Gli1, and Snail1 in (A) MHCC-97H and (C) SMMC-7721 cells treated with the miR-338-3p mimic or the negative control miRNA. **B, D.** Western blotting analysis of the relative expression levels of SMO, Gli1, and Snail1 in (B) MHCC-97H and (D) SMMC-7721 cells treated with the miR-338-3p mimic or the negative control miRNA. ***P* < 0.01.

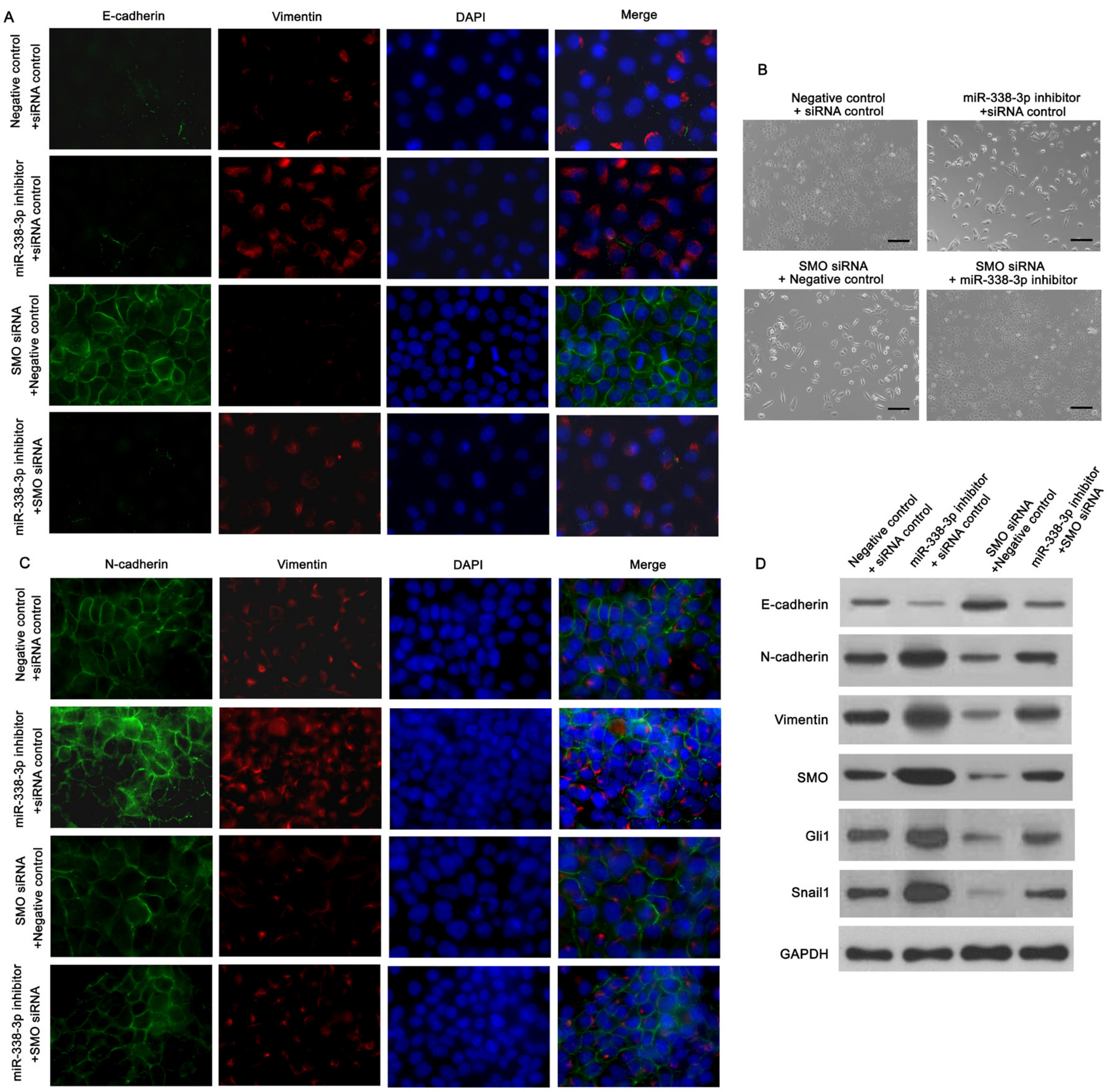

Supplementary Figure S4: Effects of the SHH pathway on miR-338-3p-inhibition-induced EMT, Snail1 expression, cell migration, and invasion. A. Single and merged images were taken to indicate immunofluorescence staining of E-cadherin (green) and vimentin (red) accompanied by the cell nucleus (blue) stained by DAPI. Original magnification, 400×. **B.** SMMC-7721 cells treated with or without miR-338-3p inhibitor or negative control, SMO siRNA or control siRNA for 24 h. Morphological changes in SMMC-7721 cells are shown by phase-contrast microscopy. Original magnification, 100×. **C.** Single and merged images were taken to indicate immunofluorescence staining of N-cadherin (green) and vimentin (red) accompanied by the cell nucleus (blue) stained by DAPI. Original magnification, 400×. **D.** Expression of E-cadherin, N-cadherin, vimentin, Gli1, and Snail1 in SMMC-7721 cells was examined by Western blot analysis. GAPDH was used as a loading control. $^{**}p < 0.01$. Scale bars: 100 µm.

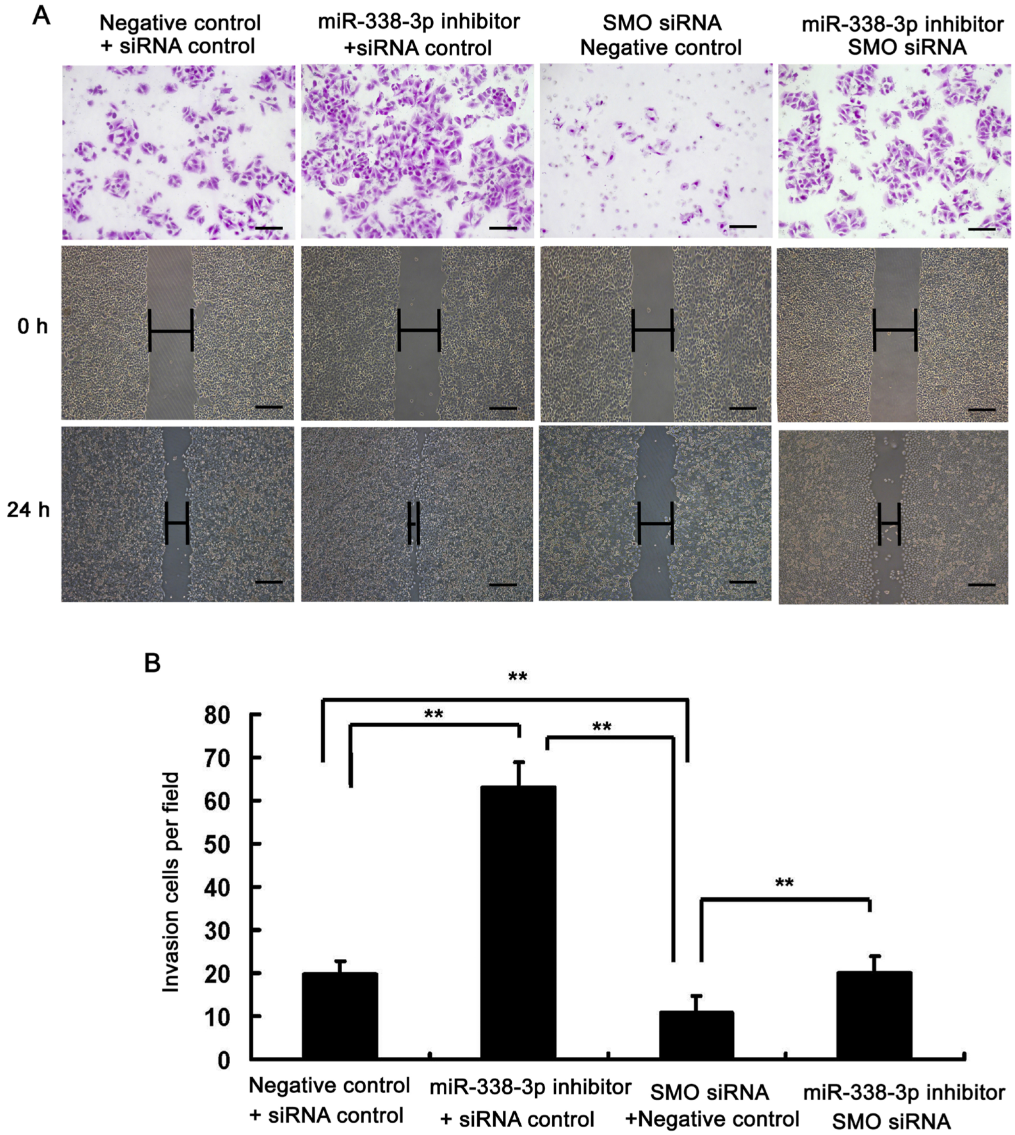

Supplementary Figure S5: Inhibition of the SHH pathway decreased miR-338-3p-induced cell migration and invasion. **A, B.** Quantification of indicated invading cells as analyzed by Matrigel-coated Transwell assays. Wound healing assay of SMMC-7721 cells transfected with miR-338-3p inhibitor or SMO siRNA. Representative pictures of one field at the beginning ($t = 0$) and at the end of the recording ($t = 48$ h) in each condition are shown. Original magnification, 200×. Scale bars: 100 µm.

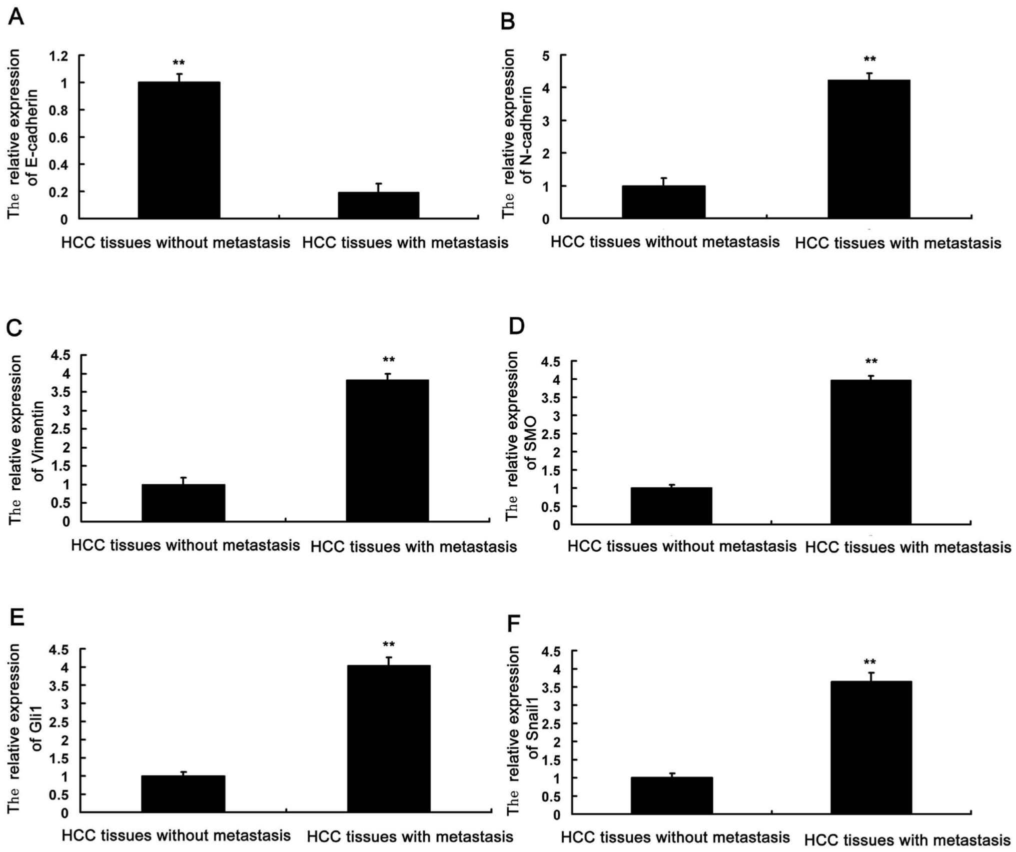

Supplementary Figure S6: Relative levels of some proteins in HCC patients with or without metastasis. A. E-cadherin, **B.** N-cadherin, **C.** vimentin, **D.** SMO, **E.** Gli1 and **F.** Snail1 The results represent the means ± SD of experiments performed in triplicate. $**P < 0.01$.

Supplementary Table S1: Clinicopathologic Variables of HCC patients

Variable	Number of case
Age	
≤50	76
>50	87
Sex	
Male	135
Female	28
Etiology	
Non-infection	19
Hepatitis B	142
Hepatitis C and other	2
Liver cirrhosis	
Absence	38
Presence	125
Tumor size (cm)	
≤5	46
>5	117
Serum AFP (μg/l)	
≤20	48
>20	115
Intrahepatic metastasis	
Absence	126
Presence	37

Supplementary Table S2: All primers and all RNA oligoribonucleotides

Name	``1
hsa-miR-338-3p mimic	5'-UCCAGCAUCAGUGAUUUUGUUG-3'<br>3'-AGGUCGUAGUCACUAAAACAAC-5'
mimics Negative control	5’-UUUGUACUACACAAAAGUACUG-3'<br>3'-AAACAUGAUGUGUUUUCAUGAC-5'
miR-338-3p inhibitor	5'-CAACAAAAUCACUGAUGCUGGA-3'
inhibitor Negative control	5'-CAGUACUUUUGUGUAGUACAAA-3'
Snail1 siRNA	5'-TCGCGGAAGATCTTCAACTGC-3'
Control siRNA of Snail1	5'-AATGGCTGCATGCTATGTTGA-3'
SMO siRNA	5'-AAGGCCTTCTCTAAGCGGCAC-3'
Control siRNA of SMO	5'-AAGCTTCGCGCCGTAGTCTTA-3'
E-cadherin primer F	5’-CAATGCCGCCATCGCTTAC-3’
E-cadherin primer R	5’-ATGACTCCTGTGTTCCTGTTAATG-3’
N-cadherin primer F	5’-GACAATGCCCCTCAAGTGTT-3’
N-cadherin primer R	5’-CCATTAAGCCGAGTGATGGT-3’
Vimentin primer F	5’-TGAGATTGCCACCTACAGGA-3’
Vimentin primer R	5’-GAGGGAGTGAATCCAGATTAGTTT-3’
SMO primer F	5'-GAGACTCTGTCCTGCGTCATCA-3'
SMO primer R	5'-AGGCATAGGTGAGGACCACAA-3'
Gli1 primer F	5'-AGGGCTGCAGTAAAGCCTTCA-3'
Gli1 primer R	5'-CTTGACATGTTTTCGCAGCG-3'
Snail1 primer F	5′-TGCAGGACTCTAATCCAAGTTTACC-3′:
Snail1 primer R	5′-GTGGGATGGCTGCCAGC-3′
β-actin primer F	5'-GGAGAATGGCCCAGTCCTC-3'
β-actin primer R	5'-GGGCACGAAGGCTCATCAT-3'
CDH2-3´-UTR F	5'-GCTAGCacttcagggtga acttggtt-3'
CDH2-3´-UTR R	5´-GCGGCCGCtttttttttttttccagatcc-3´
SMO (lacking the 3´-UTR) primer F	5´-GGAATTCCATGGCCGCTGCCCGCCCAGC-3´
SMO (lacking the 3´-UTR) primer R	5´-CAAGCTTGTCAG AAGTCCGAGTCTGCAT-3´
